# Nanoencapsulation as a General Solution for Lyophilization of Labile Substrates

**DOI:** 10.3390/pharmaceutics13111790

**Published:** 2021-10-26

**Authors:** Girish Vallerinteavide Mavelli, Samira Sadeghi, Siddhesh Sujit Vaidya, Shik Nie Kong, Chester Lee Drum

**Affiliations:** 1 Yong Loo Lin School of Medicine, National University of Singapore, 14 Medical Drive, Singapore 117599, Singapore; mdcvamg@nus.edu.sg (G.V.M.); samira.sadeghi@u.nus.edu (S.S.); mdcsid@nus.edu.sg (S.S.V.); kong.shiknie@gmail.com (S.N.K.); 2 Genome Institute of Singapore, Agency for Science, Technology and Research (A*Star), Singapore 138672, Singapore

**Keywords:** lyophilization, freeze-drying, thermostable exoshells, tES, HRP

## Abstract

Protein macromolecules occur naturally at the nanoscale. The use of a dedicated nanoparticle as a lyophilization excipient, however, has not been reported. Because biopolymeric and lipid nanoparticles often denature protein macromolecules and commonly lack the structural rigidity to survive the freeze-drying process, we hypothesized that surrounding an individual protein substrate with a nanoscale, thermostable exoshell (tES) would prevent aggregation and protect the substrate from denaturation during freezing, sublimation, and storage. We systematically investigated the properties of tES, including secondary structure and its homogeneity, throughout the process of lyophilization and found that tES have a near 100% recovery following aqueous reconstitution. We then tested the hypothesis that tES could encapsulate a model substrate, horseradish peroxidase (HRP), using charge complementation and pH-mediated controlled assembly. HRP were encapsulated within the 8 nm internal tES aqueous cavity using a simplified loading procedure. Time-course experiments demonstrated that unprotected HRP loses 95% of activity after 1 month of lyophilized storage. After encapsulation within tES nanoparticles, 70% of HRP activity was recovered, representing a 14-fold improvement and this effect was reproducible across a range of storage temperatures. To our knowledge, these results represent the first reported use of nanoparticle encapsulation to stabilize a functional macromolecule during lyophilization. Thermostable nanoencapsulation may be a useful method for the long-term storage of labile proteins.

## 1. Introduction

Proteins are widely used in clinical care with applications stretching from diagnosis to therapeutics [1,2,3,4,5]. The global protein pharmaceutical market accounted for USD 93.14 billion in 2018 and is anticipated to reach USD 172.87 billion by 2022, with more than 300 lead molecules being approved for clinical use [6]. At high concentrations, they exhibit enhanced viscosity [7,8], opalescence [9,10], aggregation, and immunogenicity [11,12,13]. These properties challenge the long-term storage of protein therapeutics and significantly affect their marketability. Though aqueous formulations are considered for many of these expensive biopharmaceuticals, they are often subjected to physico-chemical degradation resulting in instability with limited shelf-life and reduced bioactivity. Additionally, they require specific conditions for their storage and transportation, making their distribution expensive and problematic [14,15]. Therefore, powder formulations that are structurally and functionally stable at ambient storage conditions are needed.

Traditionally, lyophilization/freeze-drying is the most convenient technique used for preparing a powder form of proteins, however current lyophilization procedures have serious limitations that affect protein bioactivities. Freezing and dehydration stresses such as ice crystal formation, increase in solute concentration, changes in ionic strength, and extremes of pH often inactivate protein function either through unfolding or altering their structure [16,17,18,19]. Common excipients like sugars or sugar alcohols can offer protection during the freeze-drying procedure [20,21,22,23], however, non-specific interactions between sugar-based stabilizers and the functional residues often impact the bioactivity of the therapeutic proteins [24,25], in addition to issues of hydrolysis and adduct formation which can affect protein stability [26,27]. Because wide ranges of protein types are not suitably addressed by current excipient technologies, novel methodologies that can stabilize complex proteins and support their prolonged storage in powder form are needed.

Rapid advancements in nanotechnology have revolutionized the medical field through the development of multifunctional nanoparticles used in both diagnosis and therapy [5,28,29]. Current nanoparticle systems such as liposomes, metal, or polymer-based nanoparticles have improved the delivery of small molecule imaging probes and drugs to a variety of target sites. However, their ability to encapsulate bioactive proteins has met with relatively little progress, owing to their incompatibility of many proteins with the organic solvents used for the synthesis of colloidal particles [30]. Overall, these nanoparticle systems are typically not conducive to freeze-drying stresses as in the absence of excipients, they may undergo loss of structural integrity and the therapeutic load [31,32].

A theoretically optimal approach to the lyophilization of protein macromolecules is to surround each protein with a protective shell which also provides internally facing, stabilizing interactions for the encapsulated substrate. To this end, we modified a protein cage from *Archaeoglobus fulgidus* to accommodate host proteins and provide charge-charge stabilization [33]. The engineered tES has an 8 nm aqueous cavity that can theoretically accommodate molecules up to a volume of ~306 nm^3^ [33,34,35]. The shell, an assembly of 24 subunits, contains four 4.5 nm surface pores, which allow for easy permeation of solutes. Further, the encapsulated proteins are protected from proteases or extreme denaturing conditions [33]. Based on all these observations, we hypothesized tES by itself can operate as an effective nanoparticle lyophilization excipient and stabilize proteins in dry form. As a proof-of-principle, we have shown the stability of freeze-dried tES encapsulated HRP (tES-HRP) in comparison to free HRP over different storage conditions. Overall, tES may serve as an effective nanotechnology platform that can overcome barriers to the long-term storage and transportation of therapeutic biologicals.

## 2. Materials and Methods

### 2.1. Expression of tES

The cloning and expression of tES were executed as described previously [33]. In general, the pRSF1b vectors carrying the tES gene were transformed into non-heat shock HIT-21 competent cells (Real Biotech Corporation, Taipei, Taiwan) and cultured on Luria-Bertani (LB) agar (Axil Scientific, Singapore) plates supplied with 25 µg/mL kanamycin (Thermo Fisher Scientific, Waltham, MA, USA). Once the positive colonies were confirmed through sequencing, the glycerol stocks were prepared and stored at −80 °C. For the expression of tES, a starter culture was prepared by inoculating 100 µL glycerol stock in 100 mL of LB broth, Terrific broth (TB; Sigma, St. Louis, MO, USA), or 2XYT (Sigma, St. Louis, MO, USA) media with 50 µg/mL kanamycin. Following overnight incubation at 37 °C, 25 mL of the starter culture was used to inoculate 1 L of the respective media and allowed to grow until an absorbance (OD600) of 0.5–0.6 was reached. Protein expression was then induced with 0.4 mM IPTG (Axil Scientific, Singapore). After 5 h of incubation at 37 °C, cells were centrifuged at 12,000 rpm for 20 min and the wet weight was measured.

### 2.2. Purification of tES and Its Subunits

The harvested cell pellets from the three different media were resuspended in lysis buffer (50 mM Tris-HCl, 200 mM NaCl, 0.1% Triton-X 100 pH 8.0), sonicated, and centrifuged to obtain the lysate. The lysate was heated at 70 °C for 10 min and the precipitated sample was centrifuged at 12,000 rpm for 20 min and size-fractionated by size-exclusion chromatography (SEC) using a Superdex 200 (Cytiva, Marlborough, MA, USA) column equilibrated with 50 mM Tris-HCl pH 8.0. The purity of tES fraction was analyzed using sodium dodecyl sulfate-polyacrylamide gel electrophoresis (SDS-PAGE). Alternatively, the SEC purified tES fractions were re-chromatographed by reverse phase-high performance liquid chromatography (RP-HPLC) on a Phenomenex C_4_ column equilibrated with 0.1% trifluoroacetic acid (TFA). The bound proteins were eluted using a linear gradient of 0–100% solvent B (80% acetonitrile in 0.1% TFA) and the elution was monitored at both 215 and 280 nm. The purity and mass of the eluted protein were determined by electrospray ionization mass spectrometry (ESI-MS) using an LCQ Fleet Ion Trap mass spectrometer (Thermo Fisher Scientific, Waltham, MA, USA). Mass analysis involved injection of 25 μL of the sample at 200 μL/min flow rate into the ion source. Sheath gas flow rate of 30, auxiliary gas flow rate of 5 at a spray voltage of 4.5 KV, and capillary temperature of 350 °C were used for ionizing the sample. The ion-trap detector was set in the positive mode with a mass/ charge range of 600–2000. Qual browser, Thermo Xcalibur software was used for analysis and Pro-mass software (Thermo Fisher Scientific, Waltham, MA, USA) was used for reconstructing the mass spectrum. The concentration of the purified shell proteins was measured on a Nanodrop (DeNovix, Wilmington, DE, USA) as per Beer–Lambert’s equation by measuring the absorbance at 280 nm and using the molar extinction coefficient of ϵ280 = 814080 M^−1^∙cm^−1^. Similarly, the tES subunits were purified from the bacterial cell pellet of TB culture using the lysis buffer (50 mM sodium acetate 0.1% Triton-X 100 pH 5.8). The protein concentration was determined as mentioned above but using the molar extinction coefficient of ϵ280 = 33920 M^−1^∙cm^−1^ for the subunits.

### 2.3. Nanoencapsulation of HRP within tES

Purified tES was mildly acidified for shell disassembly in 50 mM Tris-citrate pH 5.8. The disassembled subunits were separated in SEC using the same buffer. The collected subunits were mixed with HRP (Thermo Fisher Scientific, Waltham, MA, USA) at a 10:1 molar ratio and incubated for 30 min. The pH of the mixture was increased to 8.0 using 2 M NaOH and the assembled tES-HRP was separated from free HRP using SEC with 10 mM ammonium bicarbonate pH 8.0 buffer. The activity of the free or encapsulated HRP was quantified using the 1-step^TM^ ultra TMB (Thermo Fisher Scientific, Waltham, MA, USA) assay for peroxidase wherein, the HRP oxidizes 3,3′,5,5′-tetramethylbenzidine (TMB) substrate in the presence of hydrogen peroxide to form a blue color compound. After quenching the reaction with 2 M sulphuric acid, the absorbance was recorded at 450 nm.

### 2.4. Size Determination

Dynamic light scattering (DLS) measurements were performed at room temperature (RT) using a Zetasizer Pro (Malvern Instruments Ltd., Malvern, UK). All the protein solutions used were syringe filtered by using Minisart 0.2 μm syringe filters (Sartorius, Göttingen, Germany). Stock samples of individual proteins (tES, tES-HRP, and HRP) at final concentrations of 1 mg/mL were prepared in 50 mM Tris-HCl pH 8.0. The protein concentrations were optimized in preliminary experiments to obtain reliable measurements. The ZS Xplorer software suite (Malvern Instruments Ltd., Malvern, UK) was used to analyze the acquired correlation function and to derive the translational diffusion coefficient (D). Assuming particle sphericity, the hydrodynamic diameter (d_H_) of the diffusing particles was calculated using the Stokes–Einstein equation: d_H_ = kT/3πηD where k is Boltzmann’s constant, T is the absolute temperature, and η is the viscosity of the solvent.

### 2.5. CD Spectroscopy

Far-UV CD spectra (260–190 nm) were acquired using a Jasco J-1100 CD spectrometer (Jasco, Tokyo, Japan). Protein samples (tES and HRP) at 0.2 mg/mL were prepared in 10 mM phosphate buffer and measurements were carried out at RT using a 1 mm stoppered quartz cuvette. Each spectrum was obtained after averaging three scans of the spectral range (Spectra Manager, Jasco, Tokyo, Japan). Spectra of buffer blanks were measured prior to the samples and were subtracted from the sample CD spectra.

### 2.6. Lyophilization Experiments

Before lyophilization, all the proteins (tES, tES-HRP, and HRP) were chromatographed on an SEC column equilibrated with 10 mM ammonium bicarbonate pH 8.0 and frozen at −80 °C with an exception of tES subunits that were frozen in 10 mM ammonium formate pH 5.8 [36]. They were subjected to freeze-drying for 48 h using an Alpha 1-2 LDplus lyophilizer (Christ, Osterode am Harz, Germany) at a condenser temperature of −52 °C and a vacuum pressure of 1.0 mbar. Lyophilized protein powders of tES or its subunits were stored at RT to evaluate their stability. On days 1 and 30, the reconstituted tES samples (1 mg/mL) were analyzed on SEC, pH 8.0 to assess their ability to elute as shells, considering pre-lyophilized samples as the control. Likewise, the tES subunits after 30-day storage were reconstituted at pH 8.0 or 5.8 to analyze their ability to elute as shells or subunits on SEC, respectively. Further, the dry powder of tES-HRP (10 mg/mL) or free HRP (equivalent to the activity of 10 mg/mL tES-HRP) were incubated at RT, 4 °C, and −20 °C for up to 1 month after which they were reconstituted in 10 mM ammonium bicarbonate buffer. The HRP activity was quantified using TMB assay as described previously and the percent activity was determined considering pre-lyophilization activity as 100%. Additionally, the time-dependent activity of freeze-dried tES-HRP was determined using the TMB assay measured at 652 nm. Finally, the CD spectra were acquired to study the conformational stability of the proteins post-freeze-drying and storage. All the enzyme activity experiments were conducted independently in triplicates.

### 2.7. Thermogravimetric Analysis

The residual moisture content in the freeze-dried samples was determined using Thermogravimetric analysis (TGA) on a Discovery TGA (TA Instruments, New Castle, DE, USA). Samples from three independent lyophilized vials were heated from room temperature to 1000 °C at a rate of 10 °C/min under a continuous nitrogen purge and the results were analyzed using the Trios v4 software (TA Instruments, New Castle, DE, USA).

### 2.8. Cytotoxicity Effects

The toxicity of freeze-dried tES was evaluated on the human breast cancer cell line, MDA-MB-231 (ATCC, Manassas, VA, USA). Briefly, 0.1 × 10^6^ cells were seeded in 12-well plates and cultured overnight. The following day, cells were treated with tES (30 mg/mL) or PBS as the control. The cells were incubated for 24 h at 37 °C and the cell viability was determined using the CellTiter-Glo^®^ Luminescent Cell Viability Assay (Promega, Madison, WI, USA).

## 3. Results

### 3.1. Expression and Purification of tES

**Figure 1:**
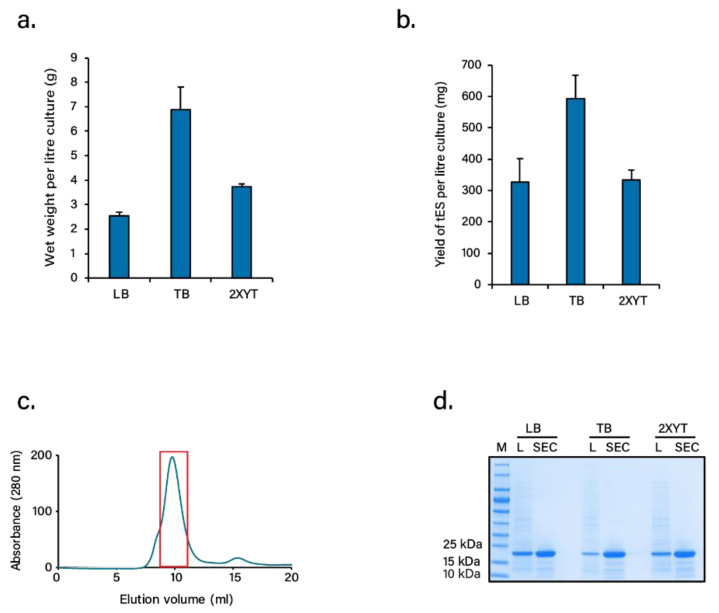
Expression of tES. (**a**) Comparison of wet weight of bacterial cell mass produced in LB, TB, and 2XYT media. (**b**) Comparison of overall yields of tES expressed in LB, TB, and 2XYT media. (**c**) Purification of tES by size-exclusion chromatography (SEC). (Note: Fractions of the tES peak (rectangle box) were analyzed for purity in SDS-PAGE, pooled, and concentrated for various experiments). (**d**) SDS-PAGE analysis of purified tES compared to lysate (L).

tES is characterized by a c-terminus truncation at Gln164 for extra internal space, internally facing mutations that create a net positive charge environment, and a Phe116His (F116H) mutation at the 3-fold symmetry axis for pH-dependent shell disassembly/assembly [33]. Acknowledging the need for high production of biomass for future use of tES as a protein excipient, we systematically compared the cell density and protein yields per liter of bacterial culture using three different media, i.e., LB, TB, or 2XYT. Of the three, TB-grown cultures demonstrated a 2–3 fold increase in cell density and protein quantity for tES compared to LB or 2XYT grown cultures, the final purified yield estimated to be ~600 mg per liter culture (Figure 1a,b). tES purification was achieved through a single-step chromatography involving SEC (Figure 1c). tES eluted around 10 mL with a minor subunit fraction around 16 mL. The purity of tES from the highlighted peak was analyzed by SDS-PAGE with a single band corresponding to a subunit mass of ~20 kDa (Figure 1d). The SEC purified fraction was further analyzed in RP-HPLC followed by ESI-MS to determine the homogeneity and mass of tES. The RP-HPLC chromatography exhibited a single peak eluted at 90% solvent B (Figure S1a, see Supplementary Materials). The protein showed multiple peaks of mass/charge (*m/z*) ratios ranging from +13 to +29 charges in ESI-MS. The observed mass of 19,286.52 Da was similar to the calculated mass of 19,287.13 Da from the amino acid sequence (Figure S1b,c, see Supplementary Materials). Additionally, the in vitro examination of purified tES was found to be non-toxic in cell viability study (Figure S2, see Supplementary Materials).

### 3.2. Lyophilization and Stability of Freeze-Dried Proteins

**Figure 2:**
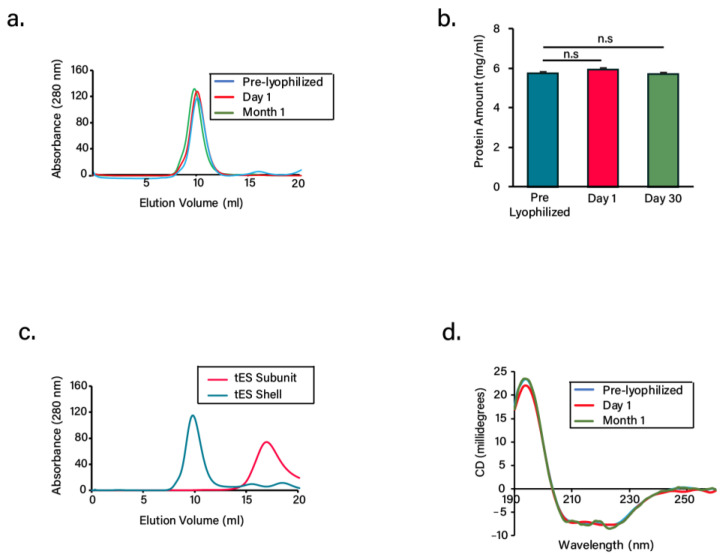
Characterization of freeze-dried tES. (**a**) Size-exclusion chromatogram of freeze-dried tES at pH 8.0 after storage at days 1 and 30 at room temperature. Pre-lyophilized samples were considered as control. (**b**) Comparison of concentrations of freeze-dried tES after storage at days 1 and 30. Pre-lyophilized samples were considered as control. (**c**) Size-exclusion chromatogram of the freeze-dried tES subunits after storage at day 30. The subunits were either reconstituted in 50 mM Tris-HCl pH 8.0 for their shell assembly or 50 mM sodium acetate buffer pH 5.8. (**d**) CD spectra of freeze-dried tES after storage at days 1 and 30 at room temperature. Compared to pre-lyophilized samples, tES exhibited double minima at 210 nm and 225 nm typical of α-helical structure (n = 3, mean ± SD).

As an attempt to understand the stability of tES or its subunits in dry powder form, we subjected the proteins to lyophilization followed by storage at RT for up to 1 month. Prior to lyophilization, the proteins were buffer exchanged to ammonium bicarbonate or formate buffer as these are readily removed during the sublimation stage [36]. The proteins after lyophilization exhibited cake shrinkage without any signs of collapse or cracking (Figure S3, see Supplementary Materials). At both day 1 and 30, the reconstituted tES co-eluted at a predicted molecular mass of 480 kDa on a calibrated SEC column, consistent with that of pre-lyophilized samples, suggesting that the shell did not disintegrate after freeze-drying and storage (Figure 2a). We also estimated the protein concentration after 1 month of storage and was found to be the same as the pre-lyophilized samples, indicating negligible protein loss (Figure 2b). Similar to tES, the subunits after their storage at RT for 1 month assembled into shells with a predicted molecular mass at pH 8.0. Alternatively, they also maintained their monomeric stature based on the elution at pH 5.8 buffer (Figure 2c). Additionally, we studied the CD spectra to observe any secondary conformational changes of the freeze-dried proteins during storage. The tES shells exhibited similar and comparable spectral patterns to the pre-lyophilized samples, with an intense minimum at 210 nm and 225 nm and maximum at 194 nm, typical of α-helical structure (Figure 2d) [37]. Taken together, the results suggest the innocuous effects of freeze-drying on the structures of tES.

### 3.3. Nanoencapsulation Studies

**Figure 3:**
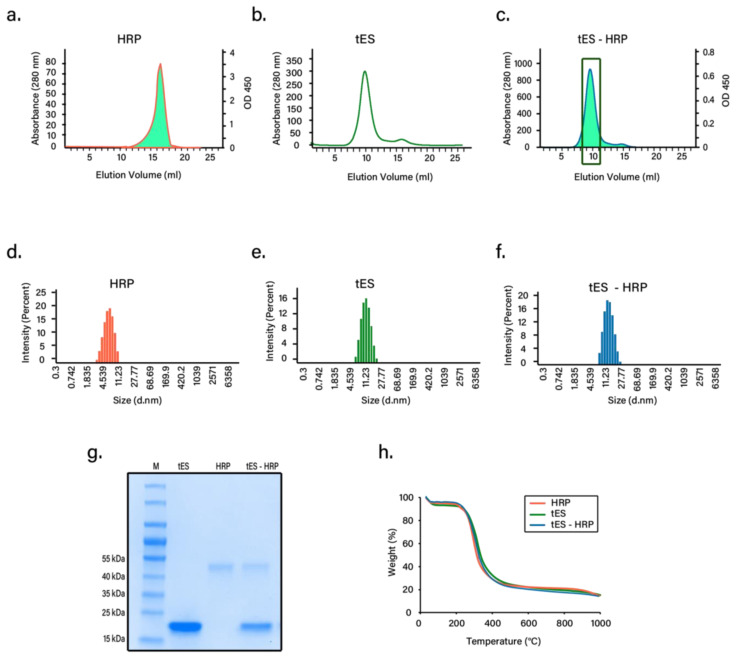
Nanoencapsulation of HRP. Size-exclusion chromatogram of free HRP (**a**), tES (**b**), and tES-HRP (**c**), at pH 8.0. Each fraction was analyzed for HRP activity and the data overlaid on the respective chromatogram (Note: Fractions of the tES-HRP peak (rectangle box) were pooled and concentrated for various experiments). Hydrodynamic diameter measurements of free HRP (**d**), tES (**e**), and tES-HRP (**f**) from DLS experiments. (**g**) SDS-PAGE analysis of nanoencapsulation. Lane 1 (M-protein marker), Lane 2 (tES) showing single band corresponding to ~20 kDa of subunit, Lane 3 (HRP) showing a single band of ~44 kDa, and Lane 4 (tES-HRP) showing two bands corresponding to subunit (~20 kDa) and HRP (~44 kDa). The quantification analysis of lane 4 through densitometry exhibited a 12-time higher intensity for tES (band intensity of 4.28 × 10^8^) compared to HRP (band intensity of 3.55 × 10^7^). (**h**) Thermogravimetric analysis (TGA) of the lyophilized proteins to determine the residual moisture content. The TGA curves of HRP, tES, and tES-HRP are shown.

Previous studies from our group have shown that HRP covalently bound to a tES subunit suitably fits inside the positive cavity of tES [33]. Based on this observation, we hypothesized that industrial HRP (Thermo Fisher Scientific, Waltham, MA, USA) could be diffusionally loaded into tES shells for lyophilization using a pH-mediated particle disassembly and assembly method. Encapsulation without any signs of aggregation was confirmed using both SEC and DLS. As expected, tES-HRP eluted as a peak at pH 8.0 with an anticipated diffusional diameter with HRP activity tracked at this peak (Figure 3a–c). Moreover, DLS displayed similar hydrodynamic diameters of 15 nm for both tES and tES-HRP confirming the internalization of HRP. The free HRP had a calculated diameter of 6 nm. All the proteins analyzed had a polydispersity index < 0.5 (Figure 3d–f). Likewise, hydrodynamic diameters were consistent with the published literature [38,39]. SDS gel electrophoresis of purified tES-HRP displayed two bands corresponding to tES subunits and HRP (Figure 3g). We determined the encapsulation of two HRP molecules within the tES and further confirmed using analytical ultra-centrifugation (unpublished observations). Further, we determined the moisture content in each freeze-dried sample using TGA. The degradation temperatures were determined from the peak of derivative weight curves. For all the samples analyzed, there were two decomposition temperatures (Td) with the first one corresponding to the water loss and the second one linked to the degradation of the protein samples, as reported earlier (Figure 3h) [40]. The average moisture content in all the freeze-dried samples is represented in Table 1.

### 3.4. Nanoencapsulation Stabilizes HRP in Dry Form

**Figure 4:**
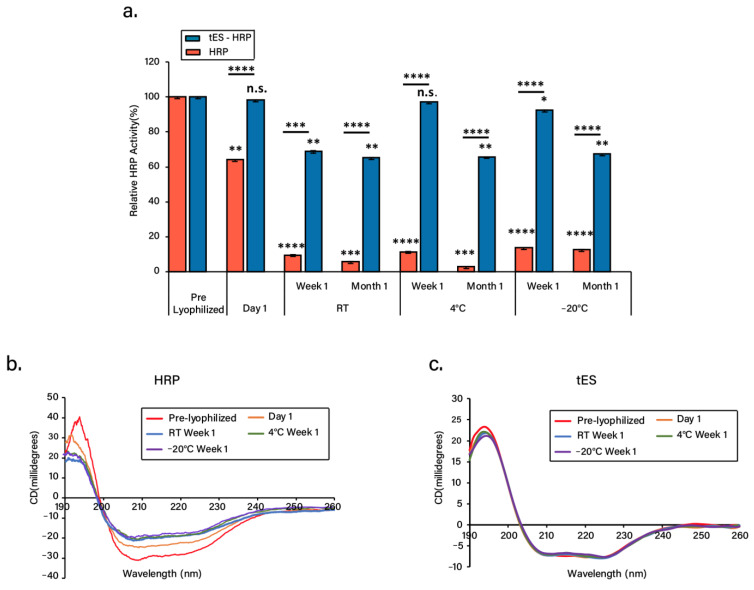
Functional and structural characterization of freeze-dried tES-HRP. (**a**) Enzyme activities of freeze-dried free or encapsulated HRP at different storage conditions: room temperature (RT), 4 °C, and −20 °C for day 1, week 1, and month 1. The enzyme activities were compared to pre-lyophilized samples (control). (**b**) CD spectra of freeze-dried HRP at different storage conditions in comparison to the pre-lyophilized sample (control). (**c**) CD spectra of freeze-dried tES at different storage conditions in comparison to the pre-lyophilized sample (control). (n = 3, mean ± SD). Statistical significance (* *p* ≤ 0.1, ** *p* ≤ 0.01, *** *p* ≤ 0.001, **** *p* ≤ 0.0001) was determined by one-way ANOVA.

The freeze-dried tES-HRP displayed significant enzyme activity even after 1 month of storage. In all the conditions evaluated, the free HRP activity was reduced to ~10% or less after 1 week/1 month storage period. Surprisingly, tES protected the HRP activity (~70–99%) under all the storage conditions evaluated (Figure 4a, Table S1). In addition, a time-course analysis of the freeze-dried tES-HRP activity was investigated (Figure S4, see Supplementary Materials). The initial velocity (v_o_) expressed as a change in absorbance per unit of time (∆A_652_/min) was determined as 0.45/min. Two important observations were made from this study. Firstly, at RT storage, tES based nanoencapsulation preserved the activity of commercially obtained HRP in spite of the required storage conditions of −20 °C. Secondly, the encapsulated HRP activity was comparable to the pre-lyophilized sample, but free HRP activity was reduced to 70% immediately after freeze-drying. To explain this, we investigated the secondary structures of HRP and tES pre and post lyophilization. The CD spectrum of pre-lyophilized HRP was characterized by a double minimum at 209 nm and 221 nm and maxima at 194 nm, indicative of a predominant α-helical structure. After freeze-drying and subsequent storage, HRP exhibited changes in the CD spectra (Figure 4b) which could be responsible for the significant reduction in the enzyme activity. A similar reduction in the enzyme activity associated with conformational changes is previously reported [41,42]. In tES, the freeze-drying had no observable effects on secondary structure as assessed by circular dichroism (Figure 4c). Collectively, these observations suggest that tES acts as a strong lyoprotectant for the encapsulated enzyme.

## 4. Discussion

The objective of lyophilization is to produce a stable formulation in dry form for long-term storage and transportation. The ability of tES to encapsulate and stabilize proteins within its aqueous cavity has previously been explored using covalently linked proteins to the internal volume and by using tES acting as a protective chaperone to support protein folding [33,43]. As an extrapolation of tES, the qualities of tES that prevent aggregation of encapsulated proteins formed the basis of the current study. HRP was selected due to its broad usage in both clinical and industrial applications such as therapeutics and diagnostics, biosensor systems, bioremediation, and biocatalysis. Additionally, the applicability of HRP in enzyme–prodrug therapy and the development of various diagnostic tools were acknowledged recently [44,45,46]. Our results demonstrate a mild pH titration protocol for protein encapsulation, wherein the tES can be disassembled at pH 5.8 and assembled at pH 8.0, a range that is expected to be compatible with the majority of protein structures [47]. Likewise, recovery of the encapsulated proteins can be achieved through mild acidification of shells [33]. HRP encapsulation was confirmed through SEC and DLS experiments (Figure 3). Furthermore, the encapsulated HRP has previously been shown to access its substrate through the 4.5 nm surface pores (Figure 4a).

The nanoencapsulation process described herein is based on complementary charge pairing wherein the negatively charged proteins such as HRP are confined within the positive shell. Similar charge-dependent encapsulations have been reported using positively charged green fluorescent protein (GFP) within the negative cavity of native archaea ferritin [48,49,50,51]. The mechanism of tES protection of encapsulated protein, we hypothesize, is two-fold. In the freezing phase of lyophilization, ice crystals are presumed to have a denaturing effect via physical disruption of protein structure. A strong, thermostable cage that surrounds the protein may thus provide a physical barrier to this denaturation. Secondly, the internal surface of the shell is engineered to complement the surface charge of the encapsulated protein. Providing this energetic stabilization may further enhance storage stability. Similar to this, hydrogen bonds are known to stabilize the dry protein formulations with the use of excipients like sugars [22,23].

A caveat of our study is that even though tES maintained HRP activity, we observed a ~30% reduction in activity over the storage period. This could be attributed to the moisture-triggered structural changes and inactivation in the freeze-dried proteins. Similar destabilization due to moisture was observed during the storage of freeze-dried therapeutic proteins like insulin and interleukin-2 [42,52]. We determined the residual moisture content in all the freeze-dried samples. TGA studies confirmed the presence of ~4–5% of residual moisture in all the samples analyzed (Table 1). Hence, it is critical to maintain the moisture content within the desired ranges as an acceptable level may ensure stabilized protein activity, non-aggregation, and easy sample reconstitution when compared to very low or high residual moisture [53,54]. Further, all the freeze-dried protein samples exhibited a classic ‘cake’ appearance with no signs of collapse or cracking (Figure S3, see Supplementary Materials). Characterized by well interconnected porous channels, a proper cake formation could be associated with maximized water removal, ease of reconstitution, or shorter freeze-drying cycles [55,56]. Despite proper lyophilization, we occasionally observed shrinkage due to the cake pulling away from the walls of the vials. Interestingly, cake shrinkage is not often associated with collapse [57]. A future direction of research may be the combination of sugar-based excipients and thermostable exoshells.

tES as a reagent is non-toxic to cell viability (Figure S2, see Supplementary Materials), can be easily purified in high yield, stored at RT for a prolonged period, and reused multiple times. In addition to high thermal stabilities (melting temperature at >90 °C), tES is extremely stable at low (−80 °C) temperatures. Thus, the dry formulations of tES encapsulated therapeutics can be advantageous in sustained release for systemic or local delivery when compared to polymeric or lipid-based systems. As tES based freeze-drying is independent of additives and homogenous on storage, they can function both as a cryoprotectant and lyoprotectant. A caveat of our study is that tES is currently limited in cargo size. For instance, encapsulation of large-size therapeutics is theoretically restricted to ~306 nm^3^. Future research is warranted to understand modifications in the protein structure to accommodate larger host proteins in addition to a detailed toxicity assessment in animal models.

## 5. Conclusions

tES demonstrated a novel freeze-drying protocol by providing a stable, immobile environment within its charged internal cavity. Further, tES retained protein activity during a one-month evaluation. tES warrants further study to understand protein recovery over prolonged periods and the mechanism of stabilization.

## Figures and Tables

**Table 1:** Decomposition temperatures (Td) and moisture content (%) of freeze-dried samples.

Sample	Td * (°C) Td ** (°C)	Moisture Content (%)
HRP	55.5 ± 2.6 307.2 ± 0.1	4.6 ± 0.2
tES	59.7 ± 2.9 328.5 ± 3.0	5.5 ± 0.9
tES-HRP	59.4 ± 3.4 329.5 ± 1.9	4.4 ± 0.8

* First decomposition temperature. ** Second decomposition temperature. All the analysis is from three independently processed samples and the values expressed as the mean ± S.D.

## Data Availability

The data presented in this study are available on request from the corresponding author.

## Supplementary Materials

The following are available online at https://www.mdpi.com/article/10.3390/pharmaceutics13111790/s1, Figure S1: Re-purification of tES. (a) RP-HPLC chromatography of SEC purified tES on a linear gradient of 0-100% solvent B. The elution was monitored at 215 and 280 nm. (b) The ESI-MS of tES showing multiple peaks of mass/charge (m/z) ratio ranging from +13 to +29 charges. (c) The mass of tES was determined to be 19286.52 Da, Figure S2: Effect of tES on cell viability, Figure S3: Cake appearance of freeze-dried proteins. (a) tES, (b) tES-HRP, (c) HRP, Figure S4: Time-dependent activity of freeze-dried tES-HRP assayed at 652 nm, Table S1: Average reads and standard deviations for HRP activity from three independent experiments.
