# Supplementary material for: Nanoencapsulation as a General Solution for Lyophilization of Labile Substrates
Source: Pharmaceutics. 2021 Oct 26;13(11):1790. doi: 10.3390/pharmaceutics13111790 (PMC8622885; doi:10.3390/pharmaceutics13111790)
Supplement: Supplementary file 1 [file pharmaceutics-13-01790-s001.zip › pharmaceutics-1368653-supplementary.pdf]

pharmaceutics

MDPI

Supplementary Materials: Nanoencapsulation as a General Solution for Lyophilization of Labile Substrates

Girish Vallerinteavide Mavelli, Samira Sadeghi, Siddhesh Sujit Vaidya, Shik Nie Kong and Chester Lee Drum

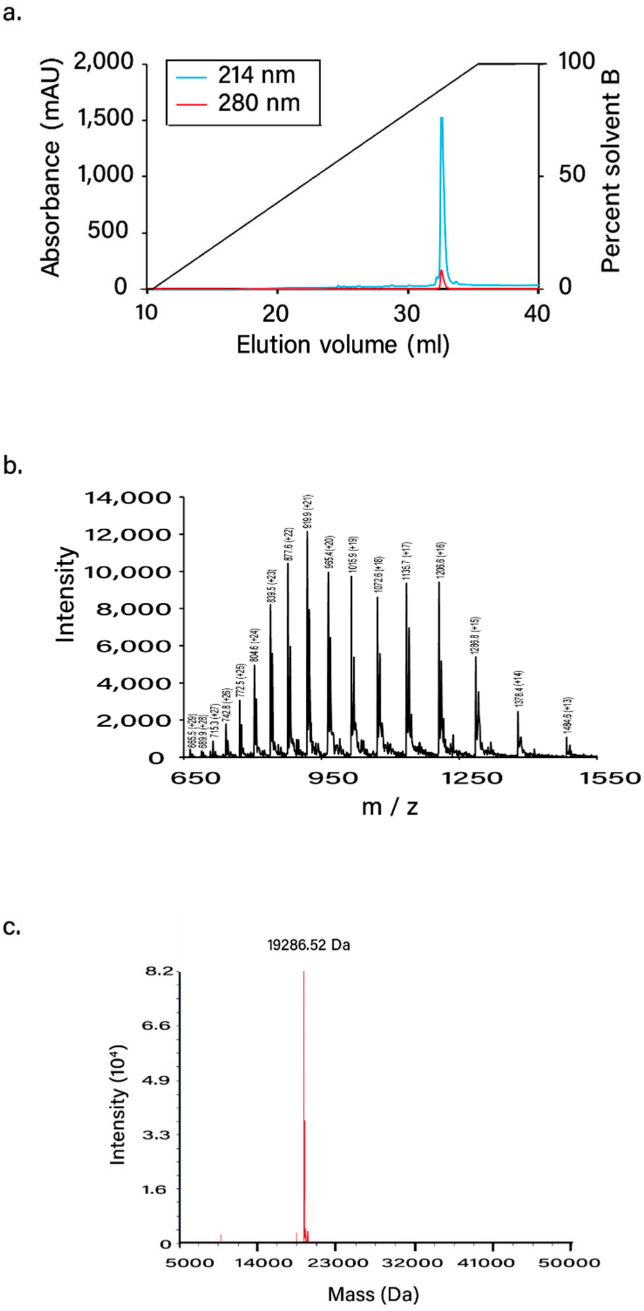

Figure S1. Re-purification of tES. (**a**) RP-HPLC chromatography of SEC purified tES on a linear gradient of 0-100% solvent B. The elution was monitored at 215 and 280 nm. (**b**) The ESI-MS of tES showing multiple peaks of mass/charge (m/z) ratio ranging from +13 to +29 charges. (**c**) The mass of tES was determined to be 19286.52 Da.

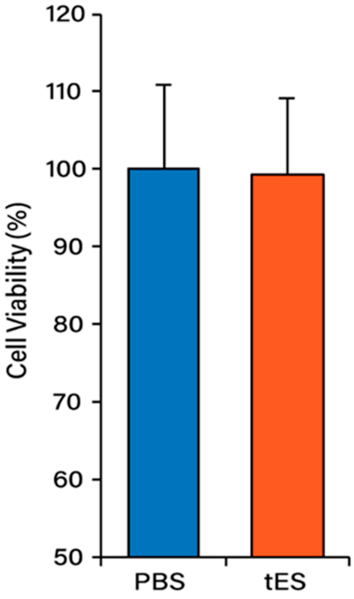

Figure S2. Effect of tES on cell viability.

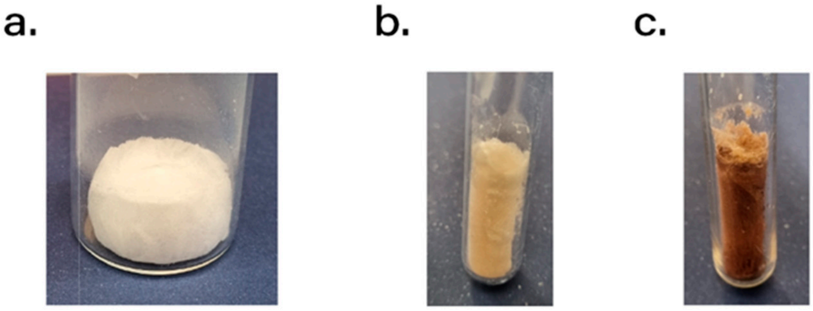

Figure S3. Cake appearance of freeze-dried proteins. (a) tES, (b) tES - HRP, (c) HRP.

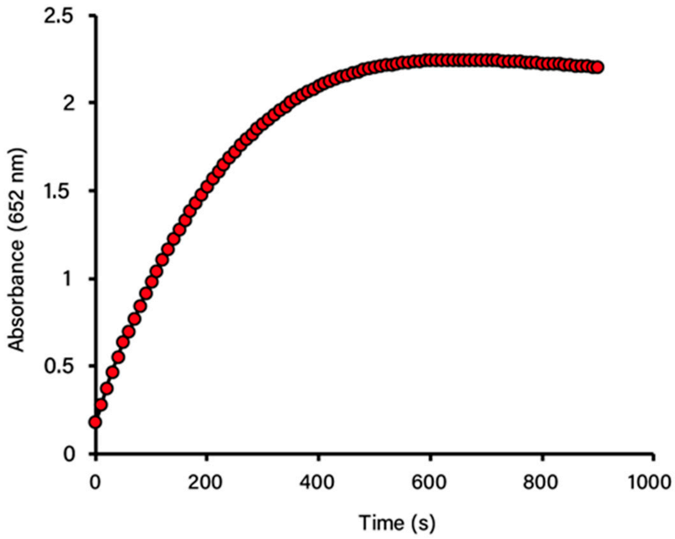

Figure S4. Time-dependent activity of freeze-dried tES-HRP assayed at 652 nm.

Table S1. Average reads and standard deviations for HRP activity from three independent experiments.

	Reads Average	STDEV	Reads Average	STDEV	Reads Average	STDEV
	**One Week**					
	RT		4 °C		-20 °C	
Lyophilized HRP	0.54	0.17	0.65	0.08	0.75	0.08
Lyophilized tES-HRP	3.92	0.27	5.53	0.18	5.22	0.13
	**One Month**					
	RT		4 °C		-20 °C	
Lyophilized HRP	0.33	0.02	0.16	0.02	0.68	0.02
Lyophilized tES-HRP	3.71	0.01	3.74	0.01	3.79	0.01
	**One Day**				**Pre-Lyophilized**	
Lyophilized HRP	3.61	0.04		HRP	5.55	0.33
Lyophilized tES-HRP	5.23	0.11		tES	5.72	0.15
